# The pepper's natural ingredient capsaicin induces autophagy blockage in prostate cancer cells

**DOI:** 10.18632/oncotarget.6415

**Published:** 2015-11-27

**Authors:** Ágata Ramos-Torres, Alicia Bort, Cecilia Morell, Nieves Rodríguez-Henche, Inés Díaz-Laviada

**Affiliations:** ^1^ Department of System Biology, Biochemistry and Molecular Biology Unit, School of Medicine, Alcala University, Alcala de Henares 28871, Madrid, Spain

**Keywords:** capsaicin, reactive oxygen species, autophagy, prostate cancer

## Abstract

Capsaicin, the pungent ingredient of red hot chili peepers, has been shown to have anti-cancer activities in several cancer cells, including prostate cancer. Several molecular mechanisms have been proposed on its chemopreventive action, including ceramide accumulation, endoplasmic reticulum stress induction and NFκB inhibition. However, the precise mechanisms by which capsaicin exerts its anti-proliferative effect in prostate cancer cells remain questionable. Herein, we have tested the involvement of autophagy on the capsaicin mechanism of action on prostate cancer LNCaP and PC-3 cells.

The results showed that capsaicin induced prostate cancer cell death in a time- and concentration-dependent manner, increased the levels of microtubule-associated protein light chain 3-II (LC3-II, a marker of autophagy) and the accumulation of the cargo protein p62 suggesting an autophagy blockage. Moreover, confocal microscopy revealed that capsaicin treatment increased lysosomes which co-localized with LC3 positive vesicles in a similar extent to that produced by the lysosomal protease inhibitors E64 and pepstatin pointing to an autophagolysosomes breakdown inhibition. Furthermore, we found that capsaicin triggered ROS generation in cells, while the levels of ROS decreased with N-acetyl-cysteine (NAC), a ROS scavenger. Co-treatment of cells with NAC and capsaicin abrogated the effects of capsaicin on autophagy and cell death. Normal prostate PNT2 and RWPE-1 cells were more resistant to capsaicin-induced cytotoxicity and did not accumulate p62 protein.

Taken together, these results suggest that ROS-mediated capsaicin-induced autophagy blockage contributes to antiproliferation in prostate cancer cells, which provides new insights into the anticancer molecular mechanism of capsaicin.

## INTRODUCTION

Prostate cancer (PC) is the second highest cause of cancer-related death among men in developed countries [1]. Risk factors associated with the leading causes of cancer death include tobacco use, overweight/obesity and physical inactivity [2]. Extensive research in the last years has collected fruitful results and European cancer mortality predictions for 2015 point to a reduction in prostate cancer-related mortality rates between 9% and 17% depending on patient's age [3]. In spite of this, cancer deaths predicted for the next years are undesirably high and a substantial portion of cancer cases and deaths could be prevented by broadly applying effective therapies. Management of early-stage prostate cancer can vary from radiation or surgery to androgen deprivation therapy [4, 5]. Unfortunately, the appearance of hormone refractory cancer cells leads eventually to the recurrence of cancer which turns to a castration resistant prostate cancer (CRPC) for which the only treatment option is chemotherapy with docetaxel. Unfortunately, patients treated with docetaxel experience significant toxicity and sometimes resistance and inevitably decline to disease progression. Therefore, a great effort is required to improve treatment options and effectiveness.

An emerging area of cancer research is focused on chemoprevention by natural compounds. Dietary products are of particular interest as chemo-preventive and chemo-therapeutic agents because of their low toxicity and potent efficacy. One of the food additive consumed worldwide is the pungent compound capsaicin which is considered a promising nutraceutical in anticancer therapy [6]. Capsaicin (8-methyl-N-vanillyl-6-noneamide) is the major ingredient of the hot chili peppers belonging to the genus *Capsicum* and responsible for their spicy flavor and burning sensation. Accumulating data have demonstrated the anti-neoplastic activity of capsaicin in many cancer cell lines as well as *in vivo* [7]. In particular capsaicin has shown anti-tumor properties against prostate cancer, inhibiting prostate tumor cells growth *in vitro* and reducing prostate growth in animal models [8, 9]. Several convergent studies have revealed that capsaicin caused cell cycle arrest and trigger apoptosis in human prostate carcinoma cells [10, 11]. Signaling mechanisms involved in capsaicin-induced prostate cell death include reactive oxygen species (ROS) generation, ceramide accumulation and NFκB inhibition [8]. In this line, we have previously shown that in prostate PC-3 cancer cells, capsaicin induces ROS generation which triggers endoplasmic reticulum stress that precedes apoptosis [12].

Endoplasmic reticulum stress accelerates the degradation of accumulated proteins within the lumen and may induce programmed cell death through activation of autophagy. Autophagy, or cellular self-digestion, is a homeostatic process where cytosolic components are targeted for removal or turnover in membrane-bound compartments (autophagosomes) that fuse with the lysosome forming the autophagolysosome. This cellular pathway is crucial for cellular fitness prolonging cell survival by recycling nutrients and energy. However, under stressful conditions sustained autophagy activation can promote cell death. Autophagy dysfunction is often associated with many diseases, including cancer, either promoting pro-survival and pro-death mechanisms depending upon the tumor type, genetic context and cellular conditions and thus, the implication of autophagy in cancer is still not completely understood. Particularly in prostate cancer, evidence of dysregulation of autophagy related proteins provide evidence that autophagy plays a relevant role in both disease progression and therapeutic resistance [13]. Therefore, targeting programmed cell death through modulation of autophagy has become a promising approach to fighting prostate cancer [14, 15]. In fact, it has been carried out autophagy-oriented clinical trials that involve autophagy modulation with therapeutic benefits [16]. In addition, natural compounds have revealed as promising agents able to modulate autophagy in prostate cancer [17].

The present manuscript examines the ability of capsaicin to trigger autophagy in prostate cancer androgen-sensitive and androgen-independent cells and the role of autophagy in capsaicin-induced cytotoxicity. A link between capsaicin-induced autophagy and ROS production has also been evaluated.

## RESULTS

### Capsaicin inhibits the PI3K/Akt/mTOR axe and modulates autophagy in both LNCaP and PC-3 cells

**Figure 1:**
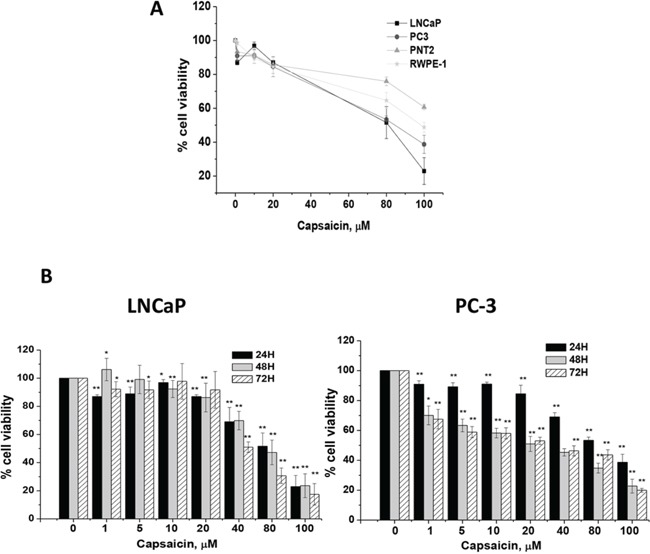
Cytotoxic effect of capsaicin on normal prostate cells and on prostate cancer cells **A.** Human normal prostate cells (PTN2 and RWPE-1) and human prostate cancer cells (LNCaP and PC-3), were treated for 24 h with different doses of capsaicin. **B.** Porstate cancer cells were treated with different doses of capsaicin for 24, 48 and 72 hours. Cell viability was monintored by MTT. Results are referred to vehicle-treated cells, considered as 100% viability. Results shown are the mean ± S.D. of four experiments performed in triplicate. *p < 0.05 and **p < 0.01 versus control compared with Student's *t* test.

We first evaluated the anti-proliferative effect of capsaicin in normal prostate PNT2 and RWPE-1 cells and in prostate cancer (LNCaP and PC-3) cells. As seen in Figure 1A, normal prostate cells were more resistant to capsaicin-induced toxicity than cancer cells. We then studied the time- and dose-dependent effect of capsaicin on prostate cancer cell lines viability. Consistent with our previous observation [10] and results from other laboratories [11] we found that capsaicin dose-dependently inhibited prostate cancer cells viability, with higher potency in the androgen-resistant PC-3 cells (IC50 =20 μM) than in the androgen-sensitive LNCaP cells (IC50 = 80 μM) (Figure 1B). Capsaicin was less effective in LNCaP cells as the anti-proliferative effect was observed at doses over 40 μM whilst in PC-3 cells a decrease in cell viability is appreciated from 1 μM capsaicin (Figure 1B). To compare the effect of capsaicin on the androgen-sensitive cells with that of the androgen-resistant cells we choose 20 μM and 80 μM doses for subsequent experiments.

**Figure 2:**
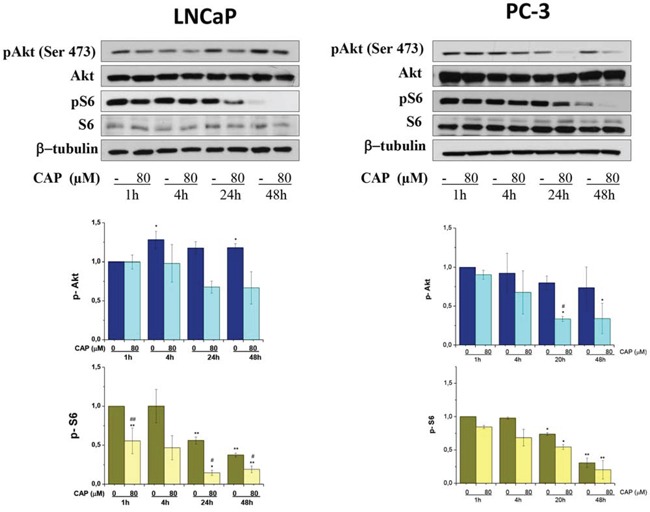
Inhibition of PI3K/Akt/mTOR axe by capsaicin in prostate cancer cells Cells were treated with vehicle (control) or 80 μM of capsaicin for different times and pAkt, Akt, pS6 and S6 proteins were detected by Western blot. Lower panel, densitometric values of the blots. Results are the mean ± S.D. of four experiments. ^#^*p* ≤ 0.05 and ^##^*p* ≤ 0.01 versus control at each concentration and *p ≤ 0.05 and **p ≤ 0.01 versus the same condition at 1 h compared with Student's *t* test.

The Akt/mammalian (or mechanistic) target of rapamycin (mTOR) signaling pathway is frequently hyperactivated in a wide assortment of human solid tumors including prostate cancer. Akt kinase mediates a potent anti-apoptotic signal in prostatic cancer and inhibition of this pathway has become an attractive mechanism to increase the efficacy of traditional chemotherapies. mTOR regulates key cellular functions downstream Akt kinase linked to the coordination of cell growth and metabolism. When active, mTORC1 triggers cell growth and proliferation by promoting protein synthesis, lipid biogenesis, metabolism and reducing autophagy. To investigate whether capsaicin impact this signaling pathway we determined the levels of phosphorylated Akt at Ser473 which is a hallmark of Akt activation. When prostate cells were incubated with capsaicin there was a time- and dose-dependent decrease in Akt phosphorylation pointing to an inhibition of this signaling pathway. mTOR activity can be monitored by following the phosphorylation of its downstream effector protein S6. Therefore, to further confirm the inhibition of Akt/mTOR axe, we measured the levels of S6 protein phosphorylation. According to the observed Akt inhibition, levels of phosphorylated S6 protein decrease in a time- and dose-dependent fashion in capsaicin-treated cells (Figure 2).

**Figure 3:**
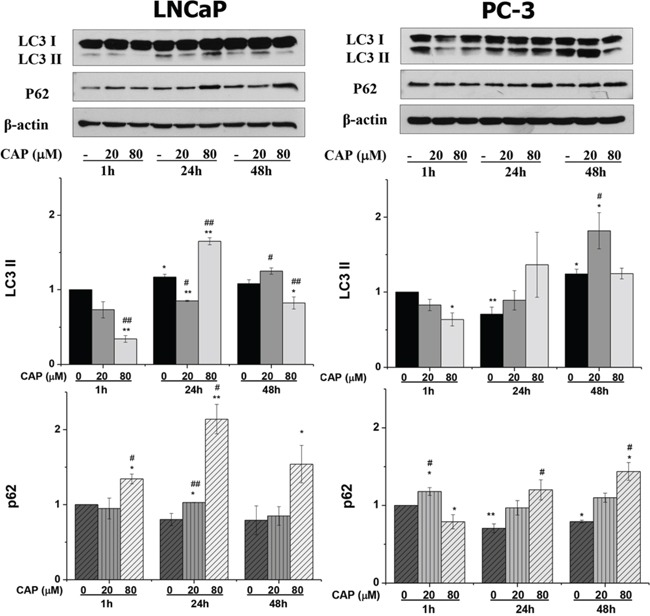
Autophagy blockage induced by capsaicin in prostate cancer cells Cells were treated with vehicle (control), 20 μM or 80 μM of capsaicin for 1 h, 24 h and 48 h and LC3 and p62 proteins were detected by Western blot. Upper panel, a representative image of four experiments. Lower panel, densitometric values of the blots. Results are the mean ± S.D. of four experiments. *p ≤ 0.05 and **p ≤ 0.01 versus control at each and ^#^*p* ≤ 0.05 and ^##^*p* ≤ 0.01 versus the same condition at 1 h compared with Student's *t* test.

A large body of literature has demonstrated that inhibition of the Akt/mTOR pathway leads to induction of autophagy in many cell types. So, we then tested whether capsaicin induced autophagy in the prostate cancer cell lines. During autophagy, microtubule-associated protein 1 light chain 3 (LC3) is cleaved at its C-terminal arginine residue to form LC3-I. LC3-I is easily activated and conjugated to phosphatidylethanolamine (PE) and bound to the autophagosome membrane to form LC3-II. This can be measured by observing the shift in molecular weight on immunoblots. To investigate whether capsaicin induced autophagy in prostate cancer cells, we determined the increase of LC3-II in capsaicin treated cells. As can be observed in figure 3, when prostate cells were treated with 20 μM and 80 μM of capsaicin an increase in the LC3-II form was observed. At 20 μM a dose a time-dependent increase in the LC3-II form can be observed. However, at 80 μM there is a peak at 24 hours treatment but at 48 hours the increase in the LC3-II form is lost. An important point is that autophagy is a dynamic, multi-step process that can be modulated at several steps, both positively and negatively. An increase in LC3-II can reflect either increased autophagosome formation due to augmented autophagic activity, or to reduced turnover of autophagosomes [18]. To further study the intensity of autophagy activity induced by capsaicin in prostate cells, we measured the levels of the p62 protein which becomes incorporated into the completed autophagosome and is degraded in autolysosomes. In addition, p62 is also required for the autophagic removal and therefore increased levels of p62 correlates with autophagy inhibition [18]. When prostate cancer cells were incubated with capsaicin for 24 h and 48 h the levels of p62 were increased (Figure 3). The increase was higher at 80 μM in both cell lines (Figure 3). These results suggest that in capsaicin-treated cells autophagy is activated but there is a blocking of autophagy that induces the accumulation of p62.

**Figure 4:**
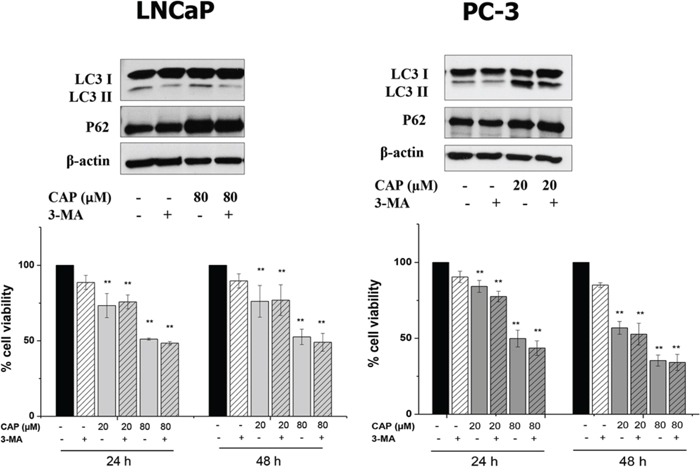
Autophagy inhibition with 3-MA do not modify the effect of capsaicin in prostate cancer cells Cells were treated with vehicle (control) or 80 μM of capsaicin in the presence or not of 1 mM 3-MA for 24 h. Upper panel and LC3 and p62 proteins were detected by Western blot. A representative image of three experiments is shown. Lower panel, viability of cells treated with vehicle (control), 20 μM or 80 μM of capsaicin in the presence or not of 3-MA for 24 h and 48 h. Results are the mean ± S.D. of four experiments. *p ≤ 0.05 and **p ≤ 0.01 versus control compared with Student's *t* test.

Interestingly, cell incubation with 3-Methyl adenine (3-MA), a class III phosphatidylinositol 3-kinase inhibitor, reduced capsaicin-induced LC3-II formation but had no impact on p62 accumulation further confirming that accumulation of p62 is due to an autophagy blocking (Figure 4). Moreover, 3-MA did not modify the anti-proliferative effect of capsaicin in prostate cells although it has an inhibitory effect when added alone (Figure 4).

**Figure 5:**
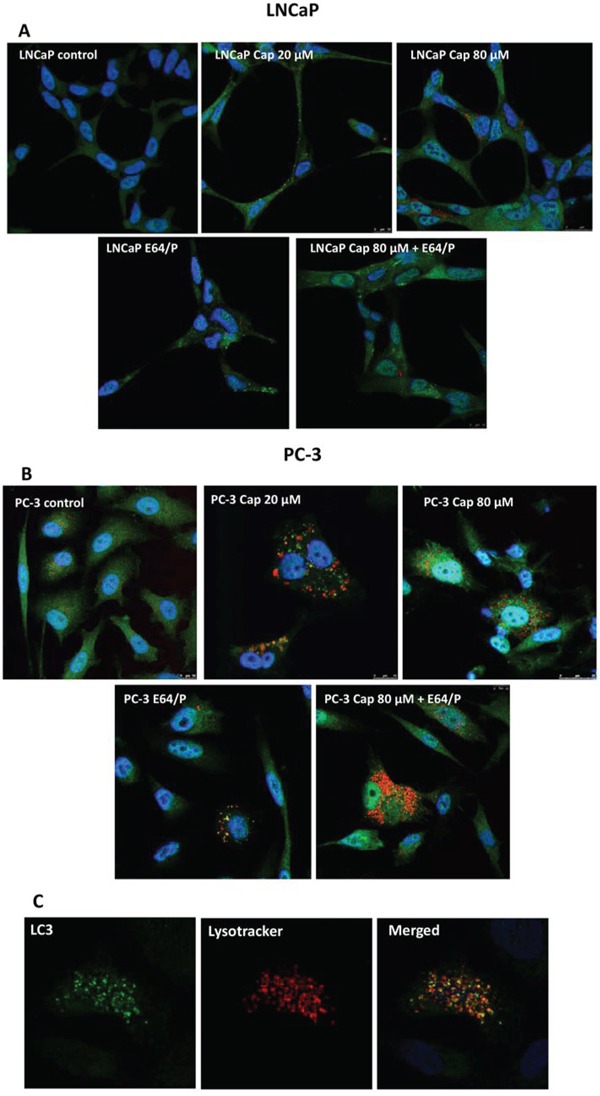
Capsaicin induces autophagy blockage in prostate cancer cells Cells were treated with vehicle (control), 20 μM or 80 μM of capsaicin for 24 h and LC3 protein was detected by immunofluorescence (green). Lysosomes were labelled with Lysotracker (red) and nuclei with DAPI (blue). **A.** LNCaP cells; **B.** PC-3 cells and **C.** magnification of PC-3 cells treated with 20 μM capsaicin. Image is representative of three experiments.

To corroborate the autophagy blocking we incubated the cells with capsaicin, labelled endogenous LC3 by inmmunofluorescence and incubated cells with Lyso Tracker Red, an acidotropic dye which primarily detect lysosomes. As shown in Figure 5A and 5B, diffuse cytoplasmic staining of LC3 was observed in non-treated control cells whereas a 50% of capsaicin-treated cells displayed punctuate LC3. In addition, capsaicin treatment increased the number of lysosomes. Interestingly, lysosomes co-localize with LC3 puncta (Figure 5C) suggesting that in capsaicin-treated cells autophagosome formation and fusion with lysosomes is activated but autophagolysosomes cannot be removed and therefore autophagy cannot flux. As a further confirmation of this result, we used the protease inhibitors E64 and pepstatin which prevent lysosomal degradation. E64 and pepstatin treatment for 30 min prior to addition of capsaicin resulted in a greater accumulation of LC3 puncta and lysosomes compared with capsaicin alone or E64+pepstatin alone (Figure 5A and 5B).

### Capsaicin treatment induces intracellular reactive oxygen species (ROS) generation

**Figure 6:**
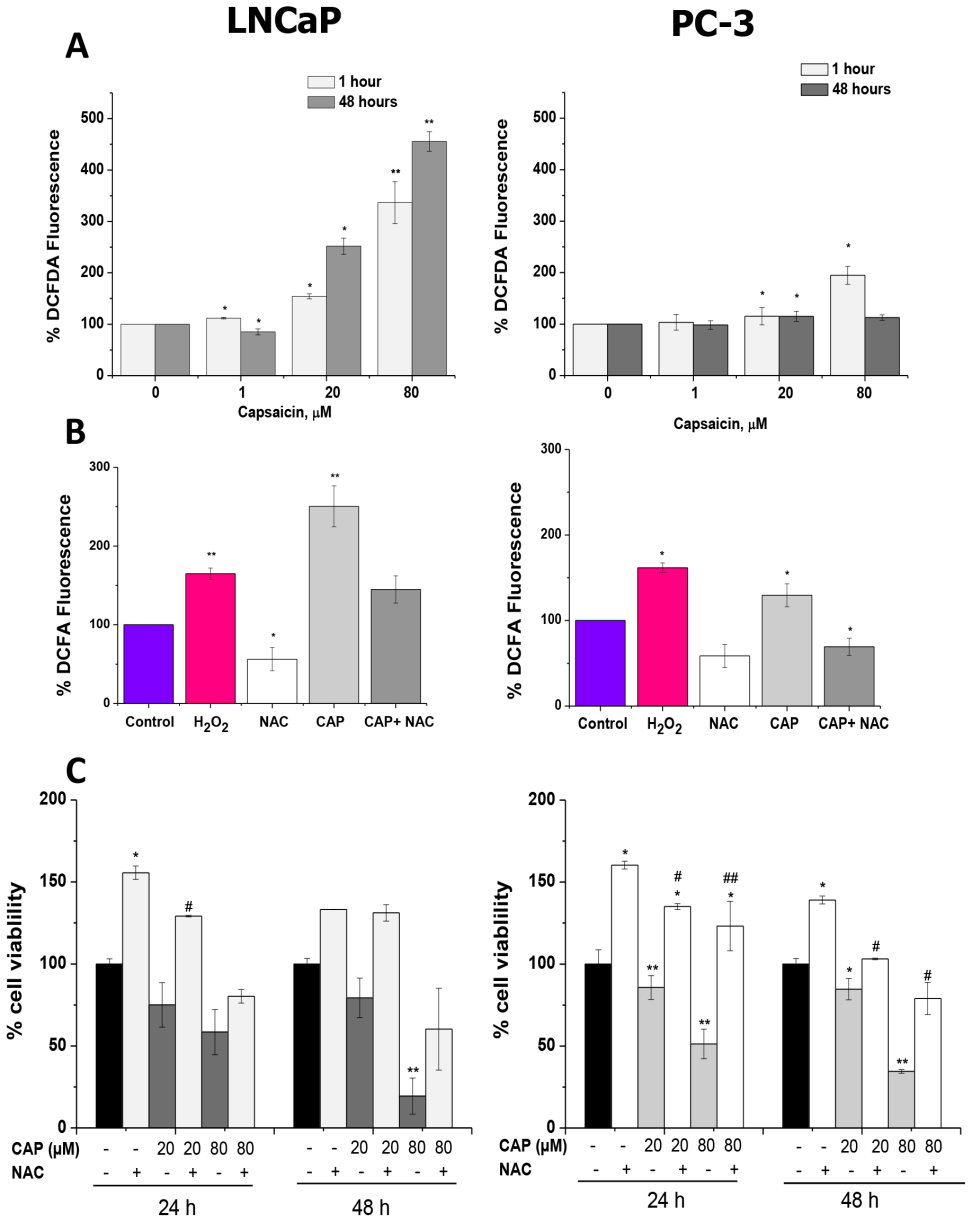
Reactive oxygen species (ROS) generation by capsaicin treatment in prostate cancer cells **A.** Cells were treated with vehicle (control), 1 μM, 20 μM or 80 μM of capsaicin for 1 h, or 48 h and ROS were measured by flow cytometry using the dye DCFDA. **B.** Cells were treated with H_2_O_2_ used as a positive control, 10 mM NAC, capsaicin 80 μM or capsaicin 80 μM and 10 mM NAC for 1 h and ROS were measured by flow cytometry. **C.** Cell viability in cells pre-treated with 10 mM NAC and then treated with 20 μM or 80 μM capsaicin for 24 h or 48 h. Results are the mean ± S.D of three experiments performed in triplicate.

To determine the effect of capsaicin on intracellular ROS production in LNCaP cells and in PC-3 cells, cells were treated with capsaicin at 1 μM, 20 μM and 80 μM for 1 hour and 48 hours. As seen in Figure 6A, in LNCaP cells, the intracellular level of ROS was significantly increased in a concentration-dependent manner. There was a fourfold increase in intracellular ROS level at 80 μM of capsaicin compared to the control LNCaP cells (*P* < 0.01). However, in PC-3 cells, capsaicin treatment did not elicit such a big increase in ROS levels. Although there was a significant twofold increase in intracellular ROS production after capsaicin treatment at 80 μM for 1 hour (*P* < 0.05; Figure 6A), at 20 μM the increase of ROS was very slight. In LNCaP cells, the increase of ROS induced by 80 μM capsaicin at 1 hour was even higher than that induced by hydrogen peroxide, used as a positive control. On the contrary, levels of ROS in capsaicin-treated PC-3 cells were lower than that induced by hydrogen peroxide (Figure 6B). In addition, co-incubation with NAC, a ROS scavenger, suppressed intracellular ROS production induced by capsaicin treatment in both LNCaP and PC-3 cells (Figure 6B).

To analyze the role of ROS on capsaicin-induced prostate cell death, we incubated the cells with 10 mM NAC prior to the treatment with of capsaicin. As shown in figure 6C, the addition of NAC increased cell viability both in control and in capsaicin-treated cells. Surprisingly, this effect was stronger in PC-3 cells than in LNCaP cells in spite of the higher ROS levels found in LNCaP cells compared with PC-3 cells.

### ROS are involved in capsaicin-induced autophagy blocking and apoptosis

**Figure 7:**
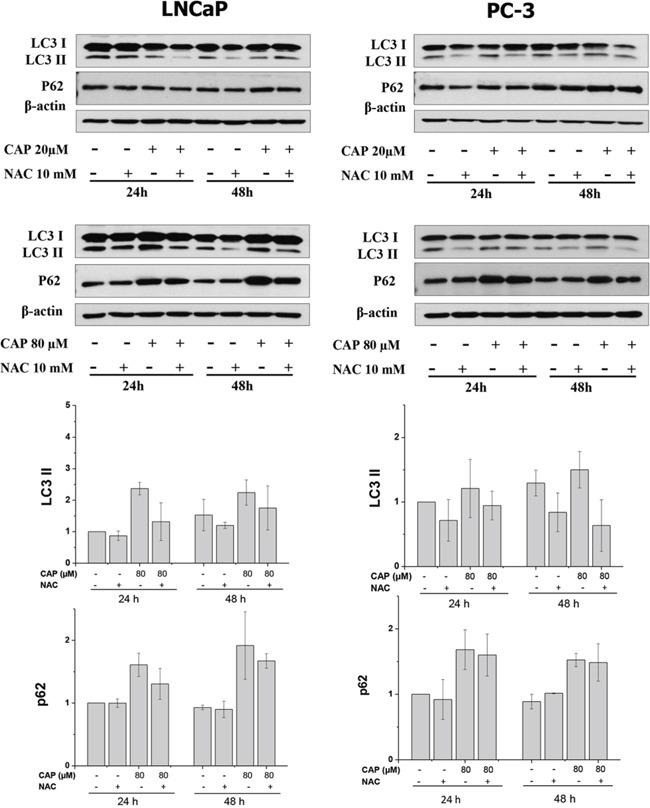
Involvement of ROS on the capsaicin-induced autophagy blockage Cells were treated with 20 μM or 80 μM of capsaicin for 24 and 48 hours in the presence or not of 10 mM *N*-Acetylcisteine (NAC). Autophagy related proteins were determined by Western Blot. Image shown is representative of other two experiments.

To further investigate the involvement of ROS on the capsaicin action on prostate cells, LNCaP and PC-3 cells were incubated with NAC prior the addition of capsaicin and autophagy marker proteins were detected by Western blot. As shown in figure 7, capsaicin-induced LC3-II conversion and p62 accumulation was decreased by NAC pretreatment. This effect was better appreciated with 80 μM of capsaicin, which induced a greater p62 increase.

**Figure 8:**
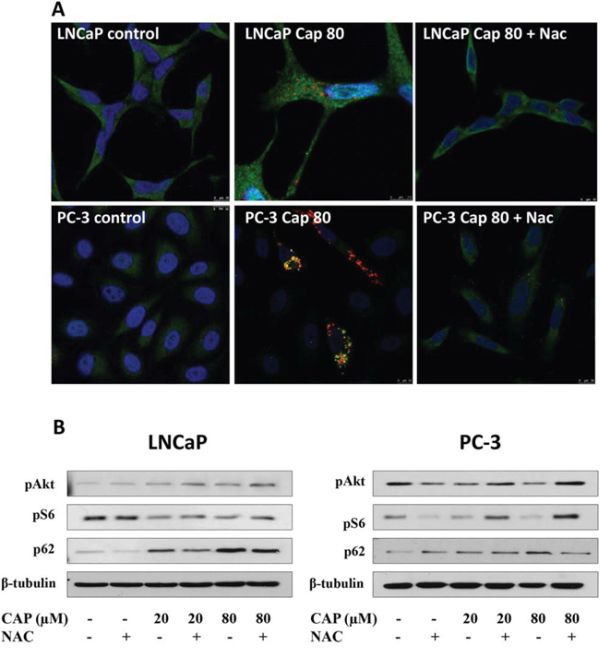
The Reactive oxygen species (ROS) scavenger NAC abrogates capsaicin-induced autophagolysosomes accumulation and Akt inhibition in prostate cancer cells **A.** Cells were treated with vehicle (control), 80 μM capsaicin or 10 mM NAC plus 80 μM capsaicin for 24 h and LC3 protein was detected by immunofluorescence (green). Lysosomes were labelled with Lysotracker (red) and nuclei with DAPI (blue). **B.** Cells were treated with vehicle (control), 20 μM or 80 μM of capsaicin for 24 h and pAkt, pS6 and p62 proteins were detected by Western blot. Image is representative of three experiments.

A stronger piece of evidence supporting the idea that NAC suppressed the autophagy blockage induced by capsaicin was obtained by confocal microscopy. When cells were incubated with capsaicin and NAC, the autophagolysosomes accumulation observed in capsaicin-treated cells was almost totally inhibited (Figure 8). Moreover, administration of NAC also reduced the inhibition of the PI3K/Akt pathway in both cell lines (Figure 8).

These data reveal that ROS play a critical role in the capsaicin-induced inhibition of PI3K/Akt and autophagolysosomes accumulation.

**Figure 9:**
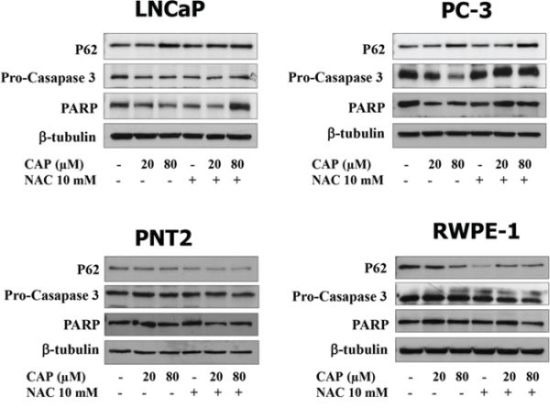
Apoptosis induction by capsaicin in prostate cancer cells but not in normal prostate cells Prostate cancer cells (LNCaP and PC-3) and normal prostate cells (PTN2 and RWPE-1) were treated with vehicle (control), 20 μM or 80 μM of capsaicin in the presence or not of *N*-Acetylcisteine (NAC) for 24 h and p62, pro-caspase 3 and PARP proteins were detected by Western blot. A representative image of three experiments is shown.

To investigate whether autophagy blockage correlated with apoptosis, prostate cells were incubated with capsaicin and levels of the caspase 3 precursor, pro-caspase 3, and poly (ADP-ribose) polymerase (PARP) were determined by Western blot. As shown in Figure 9, capsaicin dose-dependently induced a decrease in pro-caspase 3, indicating an activation of caspase 3 which plays a central role in the execution phase of cell apoptosis. Likewise, a dose-dependent decrease in PARP levels, which is a marker of apoptosis, was observed in capsaicin-treated cancer cells. Notably, the pre-treatment with NAC not only reduced the accumulation of p62 but also the reduction of pro-caspase 3 and PARP, suggesting a link between autophagy and apoptosis in the prostate cancer cells.

Those changes were not observed in the prostate normal cells PTN2 and RWPE-1 suggesting that the prostate cancer cells are more sensitive to capsaicin-induced autophagy blocking and apoptosis execution (Figure 9).

## DISCUSSION

It has been previously described that capsaicin inhibits prostate cell proliferation with different potency in the different cell lines. Based on these pre-clinical findings, interest in capsaicin is increasing in relation to the management of prostate cancer. In our study, the anti-proliferative effect of capsaicin on LNCaP and PC-3 prostate cells was confirmed by MTT assay. Results showed that capsaicin was a potent cytotoxic compound to the androgen insensitive PC-3 cells and moderately cytotoxic to the androgen sensitive LNCaP cells, whereas it exhibited a very low cytotoxicity against the normal human prostate cell lines PNT2 and RWPE-1. In addition, capsaicin induced in the prostate cancer cell lines PC-3 and LNCaP, an inhibition of the PI3K/Akt/mTOR axe which is generally upregulated in prostate cancer. As mTOR is one of the main regulators of autophagy we wondered whether capsaicin induced autophagy in prostate cancer cells. Capsaicin time-dependently increased the levels of LC3-II in both cell lines with higher potency and efficacy in PC-3 cells than in LNCaP cells.

Increased LC3 lipidation can reflect induction of autophagy, reduction in autophagosome maturation, or the inability of turnover to degrade the increased amount of autophagosomes formed [18]. Trying to discriminate between these situations, we measured levels of p62, a recruiter of ubiquitin-tagged protein aggregates to autophagosomes for specific degradation and therefore used as a cargo marker. In general, inhibition of autophagy correlates with increased levels of p62, suggesting that steady-state levels of this protein reflect the autophagic status [18]. Our results indicate that p62 accumulates in capsaicin-treated cells suggesting that breakdown of autophagolysosomes must be inhibited by capsaicin.

Confocal microscopy shows that capsaicin treatment induces an increase in the cytoplasmic LC3 puncta which co-localizes with lysosomes, which are also increased in number. This result suggest that autophagolysosome formation is greater than is its breakdown and therefore they accumulate in the cell. The mechanism through which autophagosomes fuse with lysosomes is not fully understood [19]. Recent data indicate that the class III PI3K regulator ATG14 plays a key role controlling autophagosome fusion with a lysosome [19–21]. In that study authors demonstrate that ATG14 is required for SNARE binding and autophagosome lysosome fusion promotion, but is dispensable for PI3K stimulation and autophagosome biogenesis [20]. In our study, inhibition of PI3K with 3-MA partially abrogated capsaicin-induced LC3-II increase, but did not affect p62 accumulation. This suggests that capsaicin could be acting on two points, first in the earlier steps of autophagy by inhibition of Akt/mTOR and second in the late phases of the autophagy process blocking the resolution of autophagolysosomes. Likewise, 3-MA pretreatment did not have any effect on capsaicin-induced cell death although it has an inhibitory effect when administered alone.

Autophagy is deregulated in various pathological conditions, including cancer. However the final effect of autophagy activation in cancer cells is highly variable depending on the integration of complex signaling pathways and extracellular conditions [22]. In general, autophagy activation allows cells to respond to stressful environmental conditions such as starvation or hypoxia. This function is particularly important for cancer cells that are characterized by high metabolic demand. As a prosurvival mechanism, autophagy may be used by transformed cells to adapt to the tumor microenvironment characterized by poor oxygen and nutrients supply. By other hand, as a suppressor pathway, autophagy prevents tumor initiation. Many autophagy regulators have been identified as potential cancer therapeutic agents and some cytotoxic anticancer drugs also induce autophagy [22].

Previous studies performed by other authors also demonstrate that capsaicin may induce autophagy in several malignant cell lines. Oh et al. demonstrated that Dihydrocapsaicin (DHC), a saturated structural analog of capsaicin, induced autophagy in the colorectal carcinoma cell line HCT116 as well as in the breast cancer cell line MCF-7 [23]. Interestingly, autophagy blocking with 3-MA or Atg5 siRNA, sensitized cells to apoptosis-mediated cell death [23]. The same authors also demonstrated that capsaicin and DHC triggered cell death an autophagy in WI38 normal lung epithelial fibroblast cells [24]. Studies performed by by Choi et al. showed that capsaicin induced autophagy in the human breast cancer cell lines MCF-7 and MDA-MB-231. Furthermore, capsaicin treatment in the presence of the autophagy inhibitors 3-MA or Bafilomycin A1 (BaF1) significantly reduced cell viability compared with cells treated with capsaicin only. Surprisingly, the cytotoxic effect of capsaicin was found to be much higher in normal breast epithelial MCF10A cells than in the malignant cell lines MCF-7 and MDA-MB-231, although in normal cells capsaicin did not induce autophagy [25]. This is in contrast with our results in which prostate cancer cells are more sensitive to capsaicin than normal cells. It is important to note that most of these studies show that capsaicin is effective at doses over 100 μM. It seems like capsaicin is more effective in prostate cancer cells than in other cell types as we have found that the cell viability IC50 is around 20 μM in PC-3 cells and 80 μM in LNCaP cells.

On the other hand, 80 μM capsaicin in the culture media corresponds to the dose of 1.43 mg/kg of mice body weight. Considering that most of the *in vivo* studies on the antitumoral effect of capsaicin on xenograft non prostate tumor models have used doses between 40 and 90 mg/Kg capsaicin, our results in prostate cancer cells indicate that probably lower doses will be needed to reduce prostate tumor growth. In fact, we have preliminary studies in which capsaicin exhibit antitumoral activity against prostate PC-3 cells at a dose of 25 mg/Kg/2 days [26].

Other studies have also shown that capsaicin could induce apoptosis in U251 glioma cells, and the inhibition of autophagy could contribute to apoptosis [27]. Moreover, it has been recently demonstrated that capsaicin enhances sensitivity of cholangiocarcinoma cells to 5-fluorouracil through the inhibition of autophagy [28]. These results are similar to ours and indicate that blocking of autophagy can cause cells to become sensitive to the apoptosis signaling pathway. In our study, the blockage of autophagy induced by capsaicin in prostate cancer cells may contribute to cell death as procaspase 3 and PARP are also activated.

Recent findings have shown an upregulation of autophagy proteins in prostate cancer biopsies which were highly associated with a high Gleason score and to extraprostatic invasion [29]. Therefore, it seems like in prostate cancer activation of autophagy is necessary to survival of cancer cells. In line with this notion, it has been proposed that the androgen sensitive LNCaP cells can undergo to the autophagic pathway to survive under androgen deprivation conditions, as a mechanism of transition to an androgen-independent state [30]. Current research also demonstrates that inhibition of the autophagic machinery improves prostate cell killing and tumor responsiveness [13]. In fact, autophagy inhibition is now being explored in a number of clinical trials for patients with advanced prostate cancer [13]. In this scenario, our results showing that capsaicin induces autophagy blocking in prostate cancer cells provide a potential therapeutic option for prostate cancer.

Using a cytofluorimetric approach, we have found that capsaicin induces ROS in a time and dose-dependent fashion. ROS can exert different effects according to the basal metabolic rate of the cell. The high basal metabolic rate of cancer cells makes them more susceptible to redox-directed therapeutics in comparison to non-transformed cells [31]. We have previously described that the capsaicin-induced accumulation of ROS in prostate PC-3 cells causes endothelial reticulum stress and triggers apoptosis [12]. In this study a possible relationship between capsaicin-induced ROS generation and autophagy was examined. Although connections between ROS and autophagy are observed in diverse physiological and pathological conditions, the mode of activation of autophagy and its potential protective role remain incompletely understood [32]. It is generally accepted that that ROS induce autophagy and autophagy, in turn, serves to reduce oxidative damage. In this study we found that the ROS scavenger NAC reduced capsaicin-induced LC3-II and p62 accumulation and decreased the amount of autophagolysosomes found in capsaicin-treated cells. Likewise, co-treatment with capsaicin and NAC protected against capsaicin-induced cell death and partially abrogated PI3K/Akt/mTOR inhibition. According to our study, we speculate that autophagy is necessary for prostate cell survival and that capsaicin reduces cell proliferation by a mechanism that involves autophagy blocking through ROS generation. It must be noted that although capsaicin induces an intense ROS increase in the prostate cell line LNCaP, this cell line is less sensitive to the anti-proliferative action of capsaicin as well as to the autophagy blocking. Differences in autophagy induction in both cell lines have been previously found by other authors [33]. One possible explanation is that autophagy contributes to androgen receptor (AR) degradation in LNCaP cells [34] and as capsaicin blocks autophagy, AR may be maintained at levels enough to sustain cell proliferation. This question will be addressed in future studies.

Our study underscores the importance of improving our understanding of how autophagy interacts with death pathways in transformed cells and then to use this knowledge to identify novel therapeutic targets and approaches that would more specifically treat cancer cells. One alternative therapeutic approach for prostate cancer is represented by the use of capsaicin alone or in combination with other drugs.

## MATERIALS AND METHODS

### Materials

Capsaicin (CAP), N-Acetyl cysteine (NAC) and 3-methyladenine (3-MA) were purchased to Sigma (St. Louis, USA). The inhibitors E64 and pepstatin were purchased to Roche Diagnostics (Mannheim, Germany). Primary anti-p62, anti-pAkt and antibodies were from Cell Signalling Technology (Danvers, MA, USA) and the anti-LC3 polyclonal antibody was obtained from Novus (England, UK). Peroxidase labeled secondary anti-mouse IgG was from Sigma (St. Louis, USA) and anti-rabbit IgG was from Calbiochem (San Diego, USA).

### Cell culture

Human prostate epithelial PC-3 and LNCaP cells and human prostate normal RWPE-1 cells were purchased to American Type Culture Collection (ATCC CRL-1435, CRL-1740 and CRL 11609 respectively) (Rockville, MD, USA). The human prostate normal PNT2 cell line was purchased to ECACC (European Collection of Cell cultures, Salisbury, SP4 0JG, UK). Cells were routinely grown in RPMI 1640 medium supplemented with 100 IU/ml penicillin G sodium, 100 μg/ml streptomycin sulfate, 0.25 μg/ml amphotericin B (Invitrogen, Paisley, UK) and 10% fetal calf serum. The cell line RWPE-1 was supplemented with Keratinocyte-SFM and bovine pituitary extract (0.05 mg/ml), and recombinant EGF (5 ng/ml). For treatment experiments, cells were plated and grown over night, the medium was then replaced with serum-free RPMI 1640 for 4 hours and then incubated with different doses of capsaicin for the indicated times.

### Cell viability assay

Cell viability was determined by 3-(4,5-dimethylthiazol-2-yl)-2,5-diphenyltetrazolium bromide (MTT) (Sigma-Aldrich) method. In brief, a total of 50,000 cells/well were seeded into 12-well plate in a final volume of 1mL. After treatments, 100 μl MTT solution (5 mg/ml in PBS) was added to the medium and cells were incubated at 37°C for 4 h. Then, the supernatant was discarded and dimethyl sulfoxide was added to dissolve the formazan crystals. Treatments were carried out in triplicate. The optical density in each well was evaluated by measurement of absorbance at 490 and 650 nm using a microplate reader (ELX 800 Bio-Tek Intruments, INC).

### Western blotting analysis

Cells were lysed in a lysis buffer (50 mM Tris pH 7.4, 0.8 M NaCl, 5 mM MgCl2, 0.1% Triton X-100,) containing Protease Inhibitor and Phosphatase inhibitor Cocktails (Roche, Diagnostics; Mannheim, Germany) and cleared by microcentrifugation. Protein concentrations were measured by BioRad™ protein assay kit (Richmond, CA, USA). Equal amounts of the protein samples were electrophoresed on 7%–12% sodium dodecyl sulfate polyacrylamide gel electrophoresis (SDS-PAGE) mini-gel after thermal denaturation for 5 minutes at 95°C. Proteins were transferred onto Immobilon PVDF membrane at 100 V for 2 hours at 4°C. Membranes were probed with the indicated primary antibody overnight at 4°C and then blotted with respective secondary antibody. Visualization was performed by incubation for 3 min with enhanced chemo luminescence detection buffer (100 mM Tris-HCl pH 8.5, 1.25 mM luminol, 0.2 mM p-coumaric acid, and 0.03% H_2_O_2_) and the blots were analyzed using Scion Image (Scion Corporation, Informer Technologies, Inc). The protein level was normalized to the matching densitometric value of internal control.

### Measurement of ROS

The intracellular level of ROS were analyzed with the oxidation-sensitive fluorescent probe 2′,7′-Dichlorofluorescin diacetate (DCFDA), (Sigma, St. Louis, USA). DCFDA is a cell-permeable non-fluorescent probe that is de-esterified intracellularly and turns to highly fluorescent 2′,7′-dichlorofluorescein upon oxidation and therefore provides a rapid quantitation of intracellular oxygen-reactive species. After treatments, cells were incubated with 5 μM DCFDA for 20 minutes in a 37°C incubator. Fluorescence was measured by a FACSCalibur flow cytometer. The fluorescence corresponding to the oxidized probe was followed by measuring the green (530 mm; FL1) fluorescence in the iodide propidium negative population (585 mm; FL2). Untreated cells without fluorescence were used as the background fluorescence.

### Confocal microscopy

After treatments, 100 nM lysotracker (Life Technologies, Thermo Fisher Scientific, Waltham, MA USA), was added to visualize lysosomes. Then, the cells were fixed in 4% paraformaldehyde in PBS and incubated with 0.1% Triton X-100 for permeabilization. Immunolabeling with the anti-LC3 polyclonal antibody was performed by incubation at room temperature for 1 h. Secondary labelling was performed with Alexa Flour 594, conjugated to anti-rabbit IgG and Alexa Flour 488 (Invitrogen). Imaging was performed with a Leica TCS SP5 laser-scanning confocal microscope with LAS-AF imaging software, using a 63X oil objective.

### Statistical analysis

Data were analyzed using the Student's *t* test using Origin software v 8.0 (OriginLab Corporation). Significance levels were defined as *P* < 0.05 (*,#) and *P* < 0.01 (**,##). All graphs were drawn using Origin software.

## Abbreviations

AR: androgen receptor
CAP: capsaicin
DAPI: 4′,6-diamidino-2-phenylindole
DCFDA: 2′, 7′-Dichlorofluorescin diacetate
LC3: light-chain 3
3-MA: 3-methyl adenine
NAC: N-acetyl-l-cysteine
ROS: reactive oxygen species.
